# Investigating the Appropriateness of Admission and Hospitalization at a Teaching Hospital: A Case of a Developing Country

**Published:** 2017-12

**Authors:** Mahmood NEKOEI MOGHADAM, Mohammadreza AMIRESMAILI, Reza GOUDARZI, Saeed AMINI, Sajad KHOSRAVI

**Affiliations:** 1. Health Services Management Research Center, Institute for Futures Studies in Health, Kerman University of Medical Sciences, Kerman, Iran; 2. Medical Informatics Research Center, Institute for Futures Studies in Health, Kerman University of Medical Sciences, Kerman, Iran; 3. Research Center for Modeling in Health, Institute for Futures Studies in Health, Kerman University of Medical Sciences, Kerman, Iran; 4. Health Services Management, EDC, Arak University of Medical Sciences, Arak, Iran; 5. Dept. of Health Services Management, School of Public Health, Bam University of Medical Sciences, Bam, Iran

**Keywords:** Appropriate evaluation protocol, Inappropriate admissions and hospitalization, Hospital

## Abstract

**Background:**

Reduction of inappropriate use of health services can decrease health care costs without harming the quality of services. This study aimed to investigate inappropriate admission and hospitalization at Afzalipour Hospital of Kerman, Iran.

**Methods:**

Inappropriate admission and hospitalization were assessed via a cross-sectional study on 400 patients in Kerman Afzalipour Hospital, Kerman, Iran in 2015. The Iranian version of the Appropriateness Assessment Protocol was used for data collection. Chi-square tests and logistic regression were used to analyze the data.

**Results:**

The rate of inappropriate admissions and hospitalization were 7.6% and 9% respectively. There was no significant relationship between inappropriate admissions and any of the studied variables; however, there was a significant relationship between inappropriate hospitalization and age, length of stay and hospital departments.

**Conclusion:**

If standard measures of admission and hospitalization execute correctly, unnecessary hospitalization rate can be reduced, so more patients can be treated and cost and workload of hospital personnel can be moderated.

## Introduction

Health care is one of the basic needs of each community. Since considering to healthcare and investing in this sector increases labor productivity and service production, Therefore, optimal resource allocation and use of resources is very important (1).

Evaluation of health care programs can determine their quality and progress of implementation and failure or success rate (2). Hospital services absorb almost half of health sector costs, so efficiency promotion of these services through cost reduction and use of potential capacity of health care organizations is necessary (3,4).

Diverse economic incentives have been used for cost reduction in hospitals. However, in the field of patient access to hospital services and the quality of services have not yielded to positive results (3). For preserving quality and accessibility, it is necessary to focus on cost containment indexes by attention to the appropriateness or inappropriateness of health care services (5). Some cost containment strategies such as reduction in hospital beds have increased hospital waiting time. To overcome this problem, we should use hospital beds at highest efficiency, and the best way for efficient use of hospital beds is to avoid or to minimize inappropriate patient hospitalization (6) and not to decrease the quality (7). Inappropriate hospital stays increase hospital costs, decrease available resources for patients with critical situation and put patients at risk of nosocomial infections (8). Inappropriate admission and hospitalization not only increases the costs but also cause poor health services; increase in death rate, bedsores, and blood clots because of increase in nosocomial infections. Therefore, permanent assessment of hospital services is an important issue done for improving productivity and quality of hospital services (9).

Use of hospital services for patients that the treatment had no benefit for them or the case that treatment can be delivered at less specialized levels with the same quality is called inappropriate use of hospital services (10). At the contrary, appropriate patient hospitalization is the stays that patient needs continuous and active medical, nursing and paramedical treatments and delivery of these services in other places such as day and outpatient centers cannot be performed. Some studies mainly in the American and European countries have focused on inappropriate admission and hospitalization of different patients. Inappropriate hospitalization rate varies from 6% to 78% among elderly patients, 1% to 54% among adults, 15% to 36% among neuro-surgical patients, 32% of AIDS patients, and 20% among children (11–14).

Inappropriate admission and hospitalization are one of the challenges in health sector even in developed countries. Inappropriate admission is an issue that developing countries ignore its importance and do not have any information about its severity and depth, hence, the present study aimed to investigate inappropriate admission and hospitalization at Afzalipour hospital of Kerman, Iran.

## Materials and Methods

### Study population

This study was carried out using a cross-sectional study. The study population consisted of all inpatient hospitalization at Kerman Afzalipour Hospital, Kerman, Southeastern Iran in 2015. A sample of 400 patients was selected through a stratified random sampling approach. In the first, the sample size was calculated 266 considering to the confident interval 95% (α=0.05). Then, according to studies (15), the design effect was 1.5. Therefore, the desirable sample size was calculated 400. In which four departments of infectious diseases, endocrinology, internal and gastroenterology, were selected as strata. Then we determined the sample size of each department proportional to its admission. Finally, the study subjects were selected through a simple random sampling method and there was no specific criterion for selection of samples in this study.

### Review instrument

The Iranian version of the Appropriateness Evaluation Protocol (IR-AEP) applies to the evaluation of the appropriateness of admission and hospitalization days. IR-AEP reliability and validity have been approved (16). The protocol used consists of two sections, one for admissions and the other for hospitalization days that have 24 and 31 objective criteria, respectively. If patient conditions were in accordance with at least one of the criteria, his or her admission or hospitalization was considered appropriate.

### Data collection

At the first day, the researcher visits the departments of the hospital and received list of patients that hospitalized on that day and select some patients between them randomly, then section one is completed by attention to the clinical record of the patient and appropriateness or inappropriateness of his or her admission. In the next days by researcher visit to the mentioned hospital, section two was completed for the above patients.

### Statistical analysis

The collected data were analyzed by SPSS (Chicago, IL, USA) Ver. 19 software at descriptive and analytical levels. At the descriptive level, we used frequency, percent, mean and standard deviation (SD). In order to achieve the study objectives, logistic regression test and chi-square were used. The significance level of 0.05 was adopted in all analytical tests.

## Results

Most patients were from Gastroenterology Department (131 patients). 66.5 % of the patients were male. Most patients aged 30–40 yr old. Overall, 92% of the patients had health care insurance. In addition, most patients stayed in between 6 to 10 d. The relation between demographic characteristics of the patients with inappropriate admission and hospitalization is shown in Table 1. There was no significant relation between admission and none of demographic variables. Inappropriate hospitalization had a significant relation with gender, length of stay and the type of hospitalization ward.

**Table 1:** Analytical-descriptive table of appropriate and inappropriate admission and hospitalization in relation with demographic characteristics of the patients

***Characteristics***		***Admission***			***Hospitalization***		
		**Appropriate Number (%)**	**Inappropriate Number (%)**	***P***	**Appropriate Number (%)**	**Inappropriate Number (%)**	***P***
Gender	Male	246 (92.5)	20 (7.5)	0.388	188(70.7)	78(29.3)	0.040
	Female	127 (94.8)	7 (5.2)		81(60.4)	53(39.6)	
Insurance	Yes	344 (93.7)	23 (6.3)	0.199	245(66.8)	122(33.2)	0.484
	No	29 (87.9)	4(12.1)		24(72.7)	9(27.3)	
Length of hospitalization	<5	148 (90.2)	16 (9.8)	0.149	121(73.8)	43(26.2)	0.010
	5–10	191 (95)	10 (5)		131(65.2)	70(34.8)	
	10–15	25 (100)	0 (0)		14(56)	40(25)	
	>15	9 (90)	1 (10)		3 (30)	7 (70)	
Department	Internal	102 (93.6)	7 (6.4)	0.831	71(65.1)	38(34.9)	0.010
	Gastroenterology	121(92.4)	10 (7.6)		103(78.6)	28(21.4)	
	Infectious diseases	85 (93)	7 (7)		49(53.3)	43(46.7)	
	Endocrinology	65 (95.6)	3 (4.4)		46(67.6)	22(32.4)	

In addition, in order to further explore and consider the impact of other variables, logistic regression test was used. The relation between the type of hospitalization and gender, ward and length of stay studied through logistic regression test (Table 2).

**Table 2:** The results of logistic regression test of the relation between the type of hospitalization, gender, ward and the length of stay.

***Variable***	***Multivariate Logistic regression test***					
	**B**	**SE**	***P***	**β**	**CI**	
					**Lower**	**Upper**
**Gender**						
Male(base)						
Female	0.557	0.235	0.018	1.764	1.102	2.764
**Ward name**						
Internal(base)						
Gastroenterology	−0.714	0.301	0.018	0.491	0.272	0.885
Infectious	0.533	0.30	0.076	1.703	0.945	3.070
Glands	−0.176	0.338	0.602	0.838	0.432	1.626
**Length of stay**						
<5 (base)						
5–10	0.316	0.238	0.185	1.371	0.859	2.188
10–15	0.703	0.466	0.131	2.020	0.811	5.032
>15	2.169	0.738	0.003	8.750	2.058	37.201

## Discussion

Overall, the rate of inappropriate admission in the present study was 6.7 % that is similar to the study that reported 7% inappropriate admission (17). However, it was 22.8% in a study in the selected hospitals of Tehran, different from our study (18). The studies in the American and European countries have reported inappropriate admission rate between 4% and 55%. Inappropriate admission 23% was reported in the Swedish hospitals (19). Inappropriate admission was estimated 38% in a study (20), and was reported 38.2% in Italy and 5%–45% in Spain (21). The lowest rate reported is for Neumann, so that inappropriate admission rate was 4.8% in the Turkey army hospital, which is similar to the present study (22).

In this study hospitalization days of 400 patients - that was equivalent to 2653 d- has been assessed. The findings show that 32.8% of the patients had at least one-day inappropriate hospitalization that is equivalent to 9% of all hospitalization days. In a report, inappropriate hospitalization was 8.6%, also 26.68% of the patients had at least one-day inappropriate hospitalization. In another study, inappropriate hospitalization was 6.2% (20). Majority of the studies in this issue has been from the American and Europe, so that in Villalta study 6.9% (23), in Hartz study 48% (24) and in Neumann has reported this rate 23% (22).

In this study, there was a significant relation between unnecessary hospitalization and age, Length of stay and department. By Logistic regression test, chance of inappropriate hospitalization in women was 1.76 times more than men. Gender is one of the effective factors in inappropriate admissions. Besides, to prevent job absent, men have more tendency to discharge before the specified time and women have more tendency to be hospitalized even after complete recovery. Disease incidence in women can cause more mental problems than men and because of relation between body and mental health; women have more hospitalization time than men do. In addition, female gender was a key factor to increase inappropriate hospitalization (25). In other studies, the hypothesis of gender impact on inappropriate hospitalization is not approved (17, 26). In this study the most percent of inappropriate hospitalization has been for patients with the length of stay more than 15 d. Increase in the number of hospitalization days potentially increase the number of inappropriate hospitalization days. This increase in the inappropriate hospitalization days more occurs in the last days of hospitalization. The reasons behind that are physician conservatism, patients request for more hospitalization days especially in the women, administration problems related to discharge and financial issues and absent of physicians in the discharge time. By increasing in hospitalization, the length of inappropriate hospitalization was increased (27). The most inappropriate hospitalization was for patients whose stay length was more than 15 d that is similar to the present study (17). In the present study, the difference between inappropriate hospitalizations in different departments was statistically significant; however, there was no difference between different departments (28).

Overall, difference in the inappropriate hospitalization days in different wards is related to factors like existence of many empty beds in wards, low nurse to bed proportion and internal processes such as discharge and planning surgery procedures.

## Conclusion

To achieve an effective health care system and efficient use of resources, it is necessary to eliminate unnecessary admission and hospitalization stays. Because there are factors that are out of the organization control, the aim of achieving zero unnecessary admission and hospitalization is an illusion. If standard measures of admission and hospitalization execute correctly, unnecessary hospitalization rate can be reduced, so more patients can be treated and cost and workload of hospital personnel can be moderated.

## Ethical considerations

Ethical issues (Including plagiarism, informed consent, misconduct, data fabrication and/or falsification, double publication and/or submission, redundancy, etc.) have been completely observed by the authors.

## References

[1] Arab M Zarei A Rahimi A Rezaiean F Akbari F (2010). Analysis of Factors Affecting Length of stay in Public Hospitals in Lorestan Province, Iran. Hakim Health Sys Res, 12(4): 27–32.

[2] Yaghoobi far M Maskani K Akaberi A Shahabi F (2011). The Rate of Inappropriate Admissions and Staying of Patients in Hospitals of Sabzevar, Iran. Journal of Sabzevar University of Medical Science, 18(3): 224–232.

[3] Panis G Lambert Y Frank W (2003). Predictors of inappropriate hospital stay: a clinical case study. Int J Qual Health Care, 15(1): 57–69. 12630801 10.1093/intqhc/15.1.57

[4] McDonagh MS Smith DH Goddard M (2000). Measuring appropriate use of acute beds: A systematic review of methods and results. Health Policy, 53(3): 157–184. 10996065 10.1016/s0168-8510(00)00092-0

[5] Chopard P Perneger TV Gaspoz J (1998). Predictors of inappropriate hospital days in a department of internal medicine. Int J Epidemiol, 27(3):513–9. 9698145 10.1093/ije/27.3.513

[6] Panis LJ Gooskens M Verheggen FW (2003). Predictors of in appropriate hospital stay: a clinical study. Int J Qual Health Care, 15(1): 57–65. 12630801 10.1093/intqhc/15.1.57

[7] Soria–Aledo V Carrillo–Alcaraz A Campillo–Soto A (2009). Associated factors and cost of inappropriate hospital admissions and stays in a second–level hospital. Am J Med Qual, 24(4): 321–332. 19515942 10.1177/1062860609337252

[8] Mould–Quevedo J García–Peña C Contreras–Hernández I (2010). Direct costs associated with the appropriateness of hospital stay in elderly population. BMC Health Serv Res, 9(1): 151. 10.1186/1472-6963-9-151 PMC2744673 19698130

[9] Antón P Peiró S Aranaz JM (2007). Effectiveness of a physician-oriented feedback intervention inappropriate hospital stays. J Epidemiol Community Health, 61: 128–34. 17234871 10.1136/jech.2005.040428 PMC2465655

[10] Al-Tehewy M Shehad E Al Gaafary M (2009). Appropriateness of hospital admissions in general hospitals in Egypt. East Mediterr Health J, 15(5): 1126–1134. 20214126

[11] Coast J Peters TJ Inglis A (1996). Factors Associated with inappropriate emergency hospital admission in the UK. Int J Qual Health Care, 8(1): 31–39. 8680815 10.1093/intqhc/8.1.31

[12] Levis JN Anderson GM (1996). Appropriateness in health care delivery: definitions, measurement and policy implications. CMAJ, 154: 321–328. 8564901 PMC1487507

[13] Tavakoli N Hoseini Kasnaviyeh M Yasinzadeh MR Amini M Mahmoudi Nejad M (2015). Evaluation of appropriate and inappropriate admission and hospitalization days according to appropriateness evaluation protocol (AEP). Arch Iran Med, 18(7): 430–434. 26161707

[14] Kossoovsky M Chopard P Boola F (2002). Evaluation of quality improvement intervention to reduce inappropriate hospital use. Int J Qual Health Care, 14(3): 227–232. 12108533 10.1093/oxfordjournals.intqhc.a002614

[15] Haghdoost A Baneshi M Marzban M (2011). How to Estimate the Sample Size in Special Conditions? (Part two). Irje, 7 (2): 67–74.

[16] Esmaili A Ravaghi H Seyedin H (2015). Developing of the Appropriateness Evaluation Protocol for Public Hospitals in Iran. Iran Red Crescent Med J, 17(3): e19030. 26019898 10.5812/ircmj.19030 PMC4441772

[17] Fekari A Ghiasi A Ezzati M Pakdaman M Khalafi A (2011). The Assessing of Inappropriate Admissions and Hospitalization based on Appropriate Evaluation Protocol in Alinasab hospital in Tabriz-2009. jhosp, 9(3, 4): 39–44.

[18] Pourreza A Kavosi Z Khabiri R Salimzadeh H (2008). Inappropriate admission and hospitalization in teaching hospitals of tehran university of medical sciences, Iran. Pak J Med Sci, 24(2): 301–305.

[19] Thollander J Gertow O Hansen S Carlsson B Hallert C (2004). Assessment of inappropriate emergency admissions: A study 566 consecutive cases. Lakartidningen, 101(10):888–892. 15055050

[20] Chopard P Perneger TV Gaspoz JM (1998). Predictors of inappropriate hospital days in a department of internal medicine. International Epidemiological Association, 27: 513–519. 10.1093/ije/27.3.513 9698145

[21] Young GJ Cohen BB (1992). The process and outcome of hospital care for Medicade versus privately insured hospital patient. Inquiry, 29(4):366–371. 1398905

[22] Neumann A Schultz-Coulon HJ (2001). Use of appropriateness evaluation protocol in inpatient ENT practice. HNO, 49(1): 12–20. 11219403 10.1007/s001060050702

[23] Villatla J Siso A Cereijo AC Sequeira E De Lasierra A (2004). Appropriateness of hospitalization in a short stay unit of a teaching hospital, a controlled study. Med Clin (Barc), 122(2): 454–456. 15104956 10.1016/s0025-7753(04)74270-0

[24] Hartz J Priscilla F Sigmann P. Guse C (1996 ). The evaluation of screening methods to identify medically unnecessary hospital stay for patient with Pneumonia. Int J Qual Health Care, 8: 3–11. 8680814 10.1093/intqhc/8.1.3

[25] Celik Y Celik SS Bulut HD Khan M Kisa A (2001). Inappropriate use of hospital beds: a case study of university hospitals in turkey. World Hosp Health Serv, 37(1): 6–13. 11372258

[26] Attena F Agozzino E Troisi MR Granito C Delprete U (2001). Appropriateness of admission and hospitalization days in a specialist hospital. Ann Ig, 13(2): 121–127. 11414101

[27] Appolon G Fellin G Tampieri A (1995). Appropriateness of hospital use: an overview of Italian studies. Int J Qual Health Care, 7(3):219–225. 8595458 10.1093/intqhc/7.3.219

[28] Barouni M Amini S Khosravi S (2016). Appropriateness of Delivered Services in Educational Hospitals: A Case Study in Kerman University of Medical Sciences. Sadra Med Sci J, 4(3): 185–194.

